# Extended Detrended Fluctuation Analysis of Coarse-Grained Time Series

**DOI:** 10.3390/diagnostics13010093

**Published:** 2022-12-28

**Authors:** Alexander A. Koronovskii, Inna A. Blokhina, Alexander V. Dmitrenko, Matvey A. Tuzhilkin, Tatyana V. Moiseikina, Inna V. Elizarova, Oxana V. Semyachkina-Glushkovskaya, Alexey N. Pavlov

**Affiliations:** 1 Physics of Open Systems Department, Saratov State University, Astrakhanskaya Str. 83, Saratov 410012, Russia; 2 Department of Human and Animal Physiology, Saratov State University, Astrakhanskaya Str. 83, Saratov 410012, Russia

**Keywords:** fluctuation analysis, scaling exponent, electrical brain activity, signal processing, diagnostics

## Abstract

A coarse-graining procedure, which involves averaging time series in non-overlapping windows followed by processing of the obtained multiple data sets, is the initial step in the multiscale entropy computation method. In this paper, we discuss how this procedure can be applied with other methods of time series analysis. Based on extended detrended fluctuation analysis (EDFA), we compare signal processing results for data sets with and without coarse-graining. Using the simulated data provided by the interacting nephrons model, we show how this procedure increases, up to 48%, the distinctions between local scaling exponents quantifying synchronous and asynchronous chaotic oscillations. Based on the experimental data of electrocorticograms (ECoG) of mice, an improvement in differences in local scaling exponents up to 41% and Student’s *t*-values up to 34% was revealed.

## 1. Introduction

Complex signals are usually decomposed into quite simple components to easier analyze them and understand the underlying dynamics. There are various ways to implement such a decomposition. One of them is to perform the transition from the time domain to the frequency domain using the Fourier transform and consider a set of harmonic functions whose interpretation in terms of magnitudes, frequencies, and phase shifts is clear. Another way is the Hilbert transform, which separates the amplitude and phase (or frequency) information about a non-harmonic narrowband signal and provides an approach to unambiguously determine the instantaneous amplitude and frequency. In the case of time series with several oscillatory components, an extension of this transform is applied, namely, the Hilbert–Huang transform [1], which uses decomposition of the signal into a set of empirical modes at the first stage [2]. The latter approach is a kind of filtering, where the empirical modes represent a number of filtered (i.e., simpler) signals analyzed instead of the original data.

Other variants of filtering performed within different numerical techniques can also be treated as an opportunity to simplify data analysis by introducing data sets containing reduced information about the complex structure of the signals under study. This is especially true for systems with a large number of independent or weakly interacting mechanisms involved. A typical example is the electrical activity of the brain [3], characterized by electroencephalograms (EEG), which include a number of rhythmic contributions reflecting various features of the dynamics [4]. Thus, the amplitudes of α, β, and other EEG waves make it possible to clearly characterize the electrical activity of the brain, and their study can be carried out independently. A common practice in neurophysiological researches is to separate these rhythmic contributions by means of band-pass filtering, with the cutoff frequencies of the respective filters clearly defined. The software used in such studies identifies waves of the brain electrical activity for diagnostic purposes, and this procedure simplifies the study of EEG by a transition to processes with restricted information about brain dynamics.

The choice of cutoff frequencies is motivated for the EEG, where the typical rhythmic contributions and their scale ranges are well established over a long history of neurophysiological studies and have physiological explanations. A similar analysis can be carried out for other systems in which signal processing has a background of mechanism-based studies and knowledge about the origin of rhythmic dynamics in various frequency ranges. However, even in the latter case, the distinction between independent (or almost independent) oscillatory components can be ambiguous due to incompletely separated frequency ranges, time-varying dynamics, etc. From a formal point of view, these circumstances can be ignored and signals can be processed without preliminary established filter parameters. Often this is provided within the framework of multiresolution wavelet-analysis [5] with subsequent study of signal features at different resolution levels [6,7]. Such approach is popular in physiology [8] and is also useful for other diagnostic purposes [9]. Its possible disadvantage is a dependence on the choice of the basic wavelet, i.e., on the experience of the researcher in setting up this mathematical tool for data analysis. Nevertheless, even in the case of a non-optimally selected basic function, the method can give a reliable characterization of changes in the signal structure.

Another kind of filtering has been proposed in [10], called the “coarse-graining” approach. Within this procedure, time series are averaged inside non-overlapping windows, and the resulting datasets are used for signal processing [11]. This procedure was applied to estimate the multiscale entropy, i.e., the entropy as a function of the scale parameter. However, this idea has wider applications for signal processing. It does not require selection of algorithmic parameters or prior knowledge of signal features in the frequency domain and hence the experience of the researcher does not influence the results. In contrast to “floating” window averaging, it reduces the length of the coarse-grained datasets for each window length τ as N/τ, where *N* is the length of the original time series. The aim of this study is to show that coarse-graining is able to improve the diagnostic capabilities of conventional techniques for signal processing by simultaneously analyzing data at multiple resolution levels. We explore whether the application of the coarse-graining procedure can provide a more complete characterization of complex signals compared to their processing using only the original time series. For this purpose, we compare the results of extended detrended fluctuation analysis (EDFA) proposed in [12] for the cases of signal processing with and without coarse-graining. Our research is based both on simulated data sets produced by a fairly complex model of paired nephrons [13] and on experimental data of cortical electrocorticograms (ECoG) in mice during artificial sleep.

The paper is organized as follows. Section 2 briefly describes the coarse-graining procedure and a recently proposed modification of detrended fluctuation analysis (DFA) [14] that takes into account variations in the nonstationary behavior of the time series. It also includes descriptions of the time series analyzed in this work, namely simulated datasets produced by a model of coupled functional units of the kidney and experimental recordings of ECoG in mice. Section 3 contains the main results and discussion of the comparison of EDFA for coarse-grained time series. Section 4 summarizes the concluding remarks.

## 2. Methods and Experiments

### 2.1. Coarse-Grained Time Series

For signal u(i), i=1,…,N, coarse-grained time series $v^{\tau }\left(j\right)$ are introduced according to the following procedure [15]

(1) $$\begin{array}{c} v^{\tau }\left(j\right)=\frac{1}{\tau }\sum_{i=(j-1)\tau +1}^{j\tau }u\left(i\right),\;\;\;1\leq j\leq N/\tau . \end{array}$$

This procedure means that the values u(i) are averaged inside non-overlapping time windows of duration τ. Each data point is used only once at each scale, so the created data set is significantly reduced with growing τ and contains N/τ samples.

### 2.2. EDFA Approach

The DFA method proposed by Peng et al. [14,16] and widely used in various studies [17,18,19] can be treated as a variant of signal correlation analysis when it is necessary to take into account the effects of power-law long-range correlations. According to this method, the profile of the original signal x(j),j=1,…,N

(2) $$y\left(k\right)=\sum_{j=1}^{k}\left[x(j)-\langle x\rangle \right],\;\langle x\rangle =\sum_{j=1}^{N}x\left(j\right),\;k=1,\ldots ,N$$

is introduced and it is separated into non-overlapping segments of length *n*. In this study we use coarse-grained time series $v^{\tau }\left(j\right)$ as x(j). Within each segment, the local trend $y_{n}\left(k\right)$ is fitted by the least squares algorithm. The standard deviations of the signal profile from the local trend are computed for each *n* to obtain the following relationship

(3) $$F\left(n\right)=\sqrt{\frac{1}{N}\sum_{k=1}^{N}{\left[y\left(k\right)-y_{n}\left(k\right)\right]}^{2}.}$$

In the presence of power-law correlations, this dependence has the form

(4) $$F\left(n\right)∼n^{\alpha }.$$

where the scaling exponent α can be constant over a wide range of *n* or vary with the scale depending on the signal features. In particular, the pioneering paper [16] describes the distinctions in α between short-range and long-range correlations in heart rate dynamics.

Nonstationarity affects the performance of the method, and signal preprocessing (filtering) is important to reliably characterize system behavior from experimental data [20,21,22]. Nevertheless, it is not always possible to eliminate the effects of nonstationarity at the stage of signal preprocessing. Moreover, this nonstationarity can vary throughout the signal under study. The consequence of this circumstance are different values of fluctuations of the signal profile from the trend in distinct segments. The paper [12] gives some examples when few segments make a decisive contribution to the values of F(n), and the role of the main part of the signal becomes insignificant. The latter can lead to incorrect conclusions about the underlying dynamics. To characterize the impact of nonstationarity, it was proposed to use an additional measure, namely the standard deviation of the local mean fluctuations $F_{loc}$(*n*) of the profile from the trend [23], which is often a power-law function

(5) $$\sigma \left(F_{loc}\right)\left(n\right)∼n^{\beta }.$$

The values $F_{loc}$(*n*) are evaluated for each segment of length *n*, and $\sigma \left(F_{loc}\right)\left(n\right)$ quantifies the width of the distribution of these quantities. The scaling exponent β takes negative values for processes that are close to stationary, and positive in the case of time-varying behavior of natural systems. Both α and β exponents can provide useful information for diagnosing system behavior based on experimental data.

### 2.3. Simulated Time Series

To conduct simulated studies based on rather complex mathematical models of biological oscillators, we chose a model of two interacting nephrons [13], which was proposed to describe the dynamics of paired functional units of the kidney. It was shown in [24] that this physiologically motivated model describes important features of real nephron dynamics, including bimodal oscillations, near-periodic dynamics for normal physiological states, and the transition to chaos provoked by renal hypertension [25]. In addition, the model describes various types of intra- and inter-nephron synchronization, e.g., full and partial synchronization, in-phase and anti-phase complex oscillations, etc. [26]. We consider this model as an intermediate stage between simple systems that describe the behavior of biological oscillators and natural systems. Such a consideration allows studying the complex behavior of physiological models in order to better understand the capabilities and limitations of signal processing tools for diagnostic purposes. At the same time, the simulated data sets do not contain many features of real physiological time series (random fluctuations, artifacts, various types of nonstationary dynamics, etc.).

An individual nephron produces bimodal oscillations that can be detected, e.g., in the proximal intratubular pressure or chloride concentration in the loop of Henle. Two mechanisms are responsible for such dynamics: tubular-glomerular feedback, which produces oscillations with a typical frequency of about 0.03 Hz (see experimental studies on rats [27]), interpreted as a slow mode, and the myogenic response of the afferent arteriole (fast mode with a frequency 0.1–0.2 Hz). Slow oscillations are significantly more pronounced, while the fast mode has a smaller amplitude. Because both mechanisms control the behavior of the afferent arteriole, they are not independent and affect each other, which leads to adjustment of nephron oscillations.

The single functional unit of the kidney is modeled by the following equations [13]

(6) $$\begin{array}{ccc} \frac{dP_{t}}{dt} & = & \frac{1}{C_{tub}}\left\{F_{f}\left(P_{t},r\right)-F_{reab}-\left(P_{t}-P_{d}\right)/R_{Hen}\right\},\\ \frac{dr}{dt} & = & v_{r},\\ \frac{dv_{r}}{dt} & = & \frac{1}{\omega }\left\{P_{av}\left(P_{t},r\right)-P_{eq}\left(r,\Psi (X_{3},\beta )\right)-\omega dv_{r}\right\},\\ \frac{dX_{1}}{dt} & = & \frac{1}{R_{Hen}}\left(P_{t}-P_{d}\right)-\frac{3}{T}X_{1},\\ \frac{dX_{2}}{dt} & = & \frac{3}{T}\left(X_{1}-X_{2}\right),\\ \frac{dX_{3}}{dt} & = & \frac{3}{T}\left(X_{2}-X_{3}\right). \end{array}$$

which include many nonlinearities and parameters. Due to the complex organization of the model, we do not give here its thorough description (it takes several pages and is provided in [13]). Briefly, $P_{t}$ denotes the proximal tubular pressure, $F_{f}$ is the glomerular filtration rate, $C_{tub}$ characterizes the elastic compliance of the tubule. $P_{d}$ defines the distal tubular pressure, $F_{reab}$—reabsorption, and $R_{Hen}$—flow resistance. The second and third equations simulate the arteriolar dynamics in terms of radius *r* and its rate ($v_{r}$) of changes. The parameters *d* and ω define the damping factor and the measure of relative mass. $P_{av}$ and $P_{eq}$ describe the mean arteriolar pressure and its equilibrium value; Ψ is the muscular activation. $F_{f}$, $P_{av}$, and $P_{eq}$ are computed from the numerical solution of algebraic equations. $X_{1}$, $X_{2}$, and $X_{3}$ are intermediate variables in the delay chain, and the rest of the equations describe the time delay *T* of the tubuloglomerular feedback mechanism. *T* strongly influences the dynamical regime of the nephron model. Another important parameter is the strength η of the feedback control.

The interaction of two paired nephrons can be simulated by taking two models (6) and including the coupling between them [26]. The latter is described taking into account how the activation levels $\Psi_{1,2}$ are interconnected in both units

(7) $$\Psi_{1,2}^{*}=\Psi_{1,2}+\gamma \Psi_{2,1},$$

where $\Psi_{1,2}$ are the activation levels for uncoupled, and $\Psi_{1,2}^{*}$—for coupled units, γ is the strength of coupling. We used the parameters γ= 0.005, η = 27.3. For a separate study of fast and slow oscillations in each unit, the sequences of return times into the Poincaré secant planes $P_{t}=1.6$ kPa (slow dynamics) and $v_{r}=0$ (fast dynamics) were estimated. The transition between synchronous and asynchronous chaotic oscillations was modeled by taking $T_{1}=13.5$ s, and changing $T_{2}$ from 13.5 s to 13.4 s.

### 2.4. Experimental Signals

Experimental studies were carried out on 7 male mice according to the standard Guide for the Care and Use of Laboratory Animals and the protocol approved by the Institutional Review Board of the Saratov State University (Protocol 9, 26 June 2022). The mice were housed at 25 ± 2 °C, 55% humidity, and 12:12 h light–dark cycle. Food and water were given ad libitum.

A two-channel cortical EEG (Pinnacle Technology, Taiwan) was recorded. Then, two silver electrodes (tip diameter 2–3 μm) were implanted at a depth of 150 μm in coordinates (L: 2.0 mm and P: 2 mm) from Bregma on either side of the midline under inhalation anesthesia with 1% isoflurane at 1 L/min $N_{2}O$/$O_{2}$—70:30. The head plate was mounted and small burr holes were drilled. Afterward, EEG wire leads were then inserted into the burr holes on one side of the midline between the skull and underlying dura. EEG leads were secured with dental acrylic. Ibuprofen (15 mg/kg) for the relief of postoperative pain was provided in their water supply for two to three days prior to surgery and for three days post-surgery. The animals were allowed 10 days to recover from surgery prior to beginning the experiment.

The experiments were performed in awake animals (1 h) and after anesthesia with an injection of 4% Isoflurane (i.e., under conditions of artificial sleep) (2 h). The sampling frequency was 2 kHz. The pre-processing of the signal included the removal of artifacts according to the method [28].

## 3. Results and Discussion

### 3.1. Simulated Time Series

**Figure 1:**
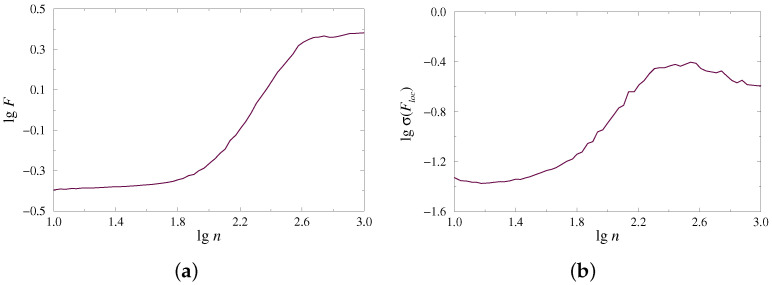
Examples of dependences F(n) (**a**) and $\sigma \left(F_{loc}\right)\left(n\right)$ (**b**) on a double logarithmic plot for the model of paired nephrons in the case of synchronous chaotic oscillations.

The complex organization of time series, which may contain multiple rhythmic contributions and various types of noise, often leads to a rather complex dependence F(n), whose characterization cannot be provided by one (global) scaling exponent. Instead, different scaling features take place for distinct ranges of scales. This circumstance is quite typical for multifractal processes requiring a spectrum of scaling exponents to describe the behavior of week and strong fluctuations. The considered example of the dynamics of paired nephrons also demonstrates multiscale dependence of lgF on lgn (Figure 1a). To perform a reliable characterization of such a dependence, the local scaling exponents can be estimated, and the latter is also valid for the second measure, which describes the power-law behavior of $\mathrm{lg}\sigma (F_{loc})$ as a function of lgn (Figure 1b).

**Figure 2:**
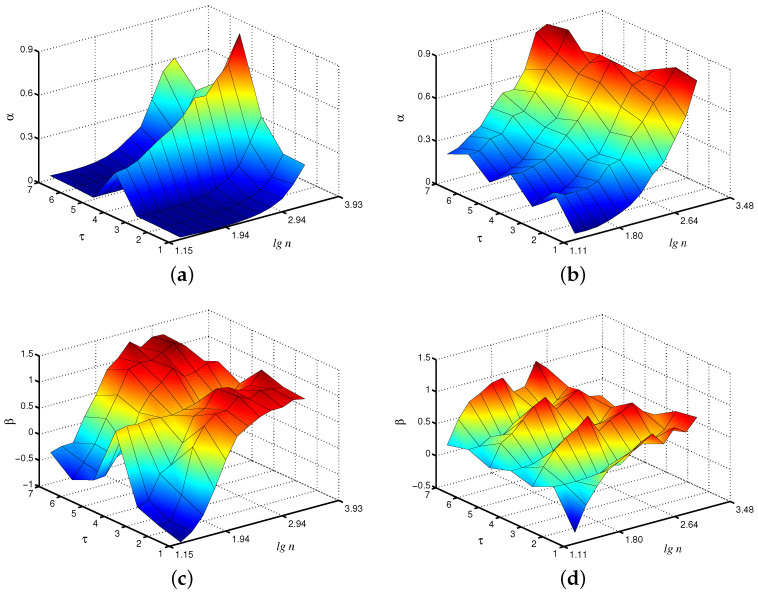
Local scaling exponents α and β for fast (**a**,**c**) and slow (**b**,**d**) oscillations in the model of paired nephrons (the case of asynchronous chaotic oscillations).

Within the framework of the considered coarse-graining procedure, which produces the time series $v^{\tau }\left(j\right)$, the EDFA algorithm can be applied to each of them. In order to better visualize the estimated quantities, we propose to show them on a 3D plot, where local scaling exponents α and β are given on a plane (τ, lgn). Let us discuss the results of this analysis for the case of fast oscillations produced by the first unit in the model of paired nephrons. According to Figure 2a, the local α-exponents vary with the scale factor τ, and the results for τ=4, e.g., are clearly different from the case τ=1 associated with the original time series. This may be explained by a kind of filtration provided within the coarse-graining [10]. Typically, slow and fast dynamics in the nephron model show 1:4 or 1:5 intra-nephron synchronization [29], and averaging over the period of the fast mode can better visualize the slow mode, which is characterized by different scaling exponents [30]. For the slow mode (Figure 2b), differences in α-exponents with a scale factor appear, but they are less pronounced. Analogous conclusions can be drawn for the β-exponent of the extended method Figure 2c,d). Again, distinctions between the cases τ=4 and τ=1 are observed in the behavior of the fast mode, but they are less expressed for the slow mode.

**Figure 3:**
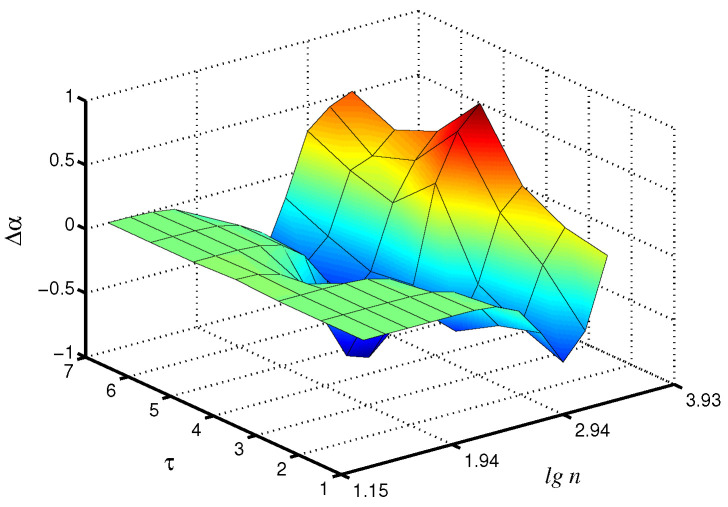
Differences Δα in local values of α for synchronous and asynchronous chaotic oscillations studied for the fast mode of the first nephron.

Changes in the control parameters of the model, leading to transitions between synchronous and asynchronous chaotic oscillations [26], affect the values of the scaling exponents. Figure 3 shows the differences Δα in the local scaling exponents between synchronous chaotic dynamics ($T_{1}=T_{2}=13.5$ s) and asynchronous chaotic oscillations ($T_{1}=13.5$ s, $T_{2}=13.4$ s) for the case of the fast mode in the first nephron, where the distinctions are more pronounced. Note that these distinctions are clearer for the scale factor τ=4, i.e., the coarse-graining procedure makes it possible to better identify changes in the signal structure than the consideration of the original time series with its conventional analysis (Δα increases by 48% for τ=4 compared to τ=1).

**Figure 4:**
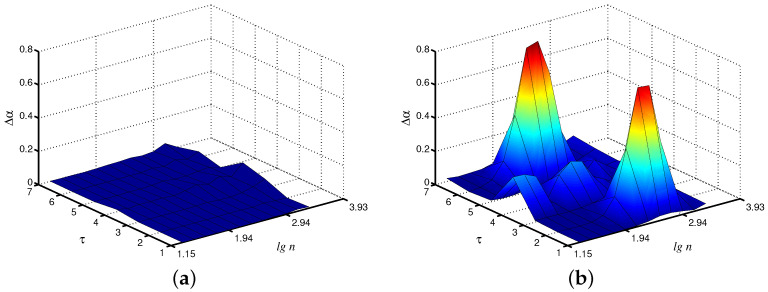
Differences Δα in local values of α for both interacting units (fast mode) for synchronous (**a**) and asynchronous (**b**) chaotic oscillations.

When dealing with the synchronization phenomenon, the diagnostics of entrainment of complex systems with self-sustained oscillations is provided by studying interacting units from related time series. In addition to adjustment of the instantaneous frequencies or phases of the oscillations, synchronization often results in the similarity of various measures of the complex dynamics of coupled subsystems [24]. With regard to the model of paired nephrons, we can state the similarity of the local scaling exponents for the fast mode in each nephron (Figure 4a). In the asynchronous case, the distinctions in the dynamics of both units are stronger (Figure 4b). Note that these distinctions are mainly observed not for the original time series, but for the coarse-grained data set with scale factors τ=2 and τ=6. Therefore, coarse-graining seems to be a useful approach to better identify distinctions in system behavior caused by the synchronization phenomenon.

### 3.2. Experimental Signals

**Figure 5:**
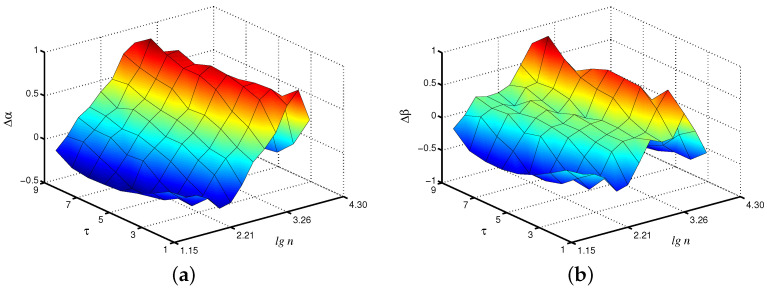
A typical example of differences Δα and Δβ in local scaling exponents α (**a**) and β (**b**) between waking and artificial sleep states. Improvements by 32% (**a**) and 41% (**b**) compared to τ=1 are observed.

The ECoG signals were analyzed by analogy with the simulated data sets. Figure 5 shows an example of differences in local scaling exponents in wakefulness and artificial sleep states for a typical animal. These results do not demonstrate clear advantages in the choice of the scale factor (Figure 5a), but they show that the consideration of the original signal (i.e., the case τ=1) does not provide the best diagnosis of emerging changes in the brain dynamics. Thus, when estimating the α exponent, we observe the strongest distinctions in the range near lgn=3.0, and these distinctions increase with the growth of τ. The use of the β exponent also leads to a similar conclusion that consideration of coarse-grained time series can be useful for characterizing the state of the system (Figure 5b).

**Figure 6:**
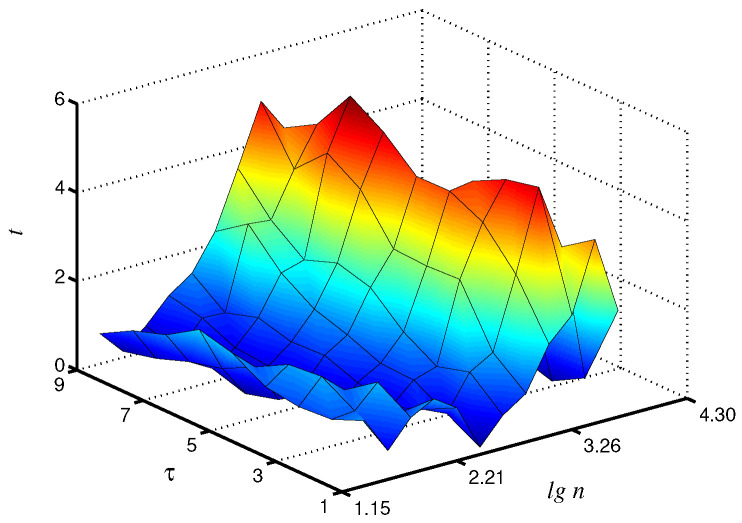
Statistical analysis of differences in local α values by Student’s *t*-test. Improvement by 34% compared to τ=1 is observed.

Statistical analysis carried out on a group of animals confirms these findings. In particular, the Student’s *t*-test provided the largest *t*-values at τ=7 for α-exponent, although significant (p<0.05) distinctions also occur in a wide range of τ and lgn (Figure 6). This exponent provided clearer inter-group differences in the considered range of lgn. For β-exponent, significant distinctions were also detected, although the obtained results are more dependent on the selection of lgn. The results of this study demonstrate that the application of the coarse-graining procedure followed by processing of the related time series can improve the analysis of complex systems from recorded time series for diagnostics purposes and the detection of changes in system dynamics.

Thus, the coarse-graining approach [10,11] is a kind of signal transformation in the frequency domain that introduces a number of data sets associated with different values of the scale factor. This approach provided an informative characterization of various types of complex signals as a part of the method for multiscale entropy computing, which showed clear advantages over traditionally used techniques dealing with scalar time series. In addition to complexity analysis, the same procedure is useful in other applications to study system behavior in more detail. It is carried out without prior knowledge of the signal features and the presented frequency components, what makes the analysis independent of the experience of the researcher, i.e., subjective factors do not essentially affect the results and conclusions.

## 4. Conclusions

In this study, we compared the results of processing both simulated and experimental data with and without coarse-graining based on the EDFA method. The advantages of this procedure over traditional data analysis can be qualitatively described by 3D presentation of the results with visual identification of changes and quantified by differences in local scaling exponents and *t*-values of the Student’s test. Using a model of two paired nephrons, it is shown that the results for coarse-grained time series can be superior to the results for the original data set, and the latter is valid for both transitions between the regimes of synchronous and asynchronous chaotic oscillations studied from time series relating to only one and both interacting subsystems. In particular, improvements in the quantification of differences in local scaling exponents of up to 48% between synchronous and asynchronous chaotic oscillations studied for the fast mode of one nephron were identified. In experimental studies of ECoG signals during wakefulness and artificial sleep, these findings were confirmed, and advantages in the detection of changes in system dynamics for coarse-grained time series were shown and verified by Student’s *t*-test. The related improvement takes up to 41% in local scaling exponents and up to 34% in *t*-values. These results confirm that the coarse-graining procedure can provide wider abilities in diagnostic-related studies compared to conventional signal processing of the original data set.

## Data Availability

The data that support the findings of this study are available from the corresponding author upon reasonable request.

## Abbreviations

The following abbreviations are used in this manuscript:

DFA	Detrended fluctuation analysis
EDFA	Extended detrended fluctuation analysis
EEG	Electroencephalogram
ECoG	Electrocorticogram
